# Brazil, Russia, China, India, and South Africa (BRICS) – Partnership for Sustainable Development and Inclusive Multilateralism: A new alternative for implementing the 1978 Alma Ata Declaration on primary health care

**DOI:** 10.7189/jogh.13.03066

**Published:** 2023-12-29

**Authors:** Guy O Kamga Wambo

**Affiliations:** Internistische Hausarztpraxis Dr. Kamga, Berlin, Germany

Throughout history, economic growth and geostrategic influences have been intertwined with a collective awareness of the importance of improving the health of populations. We can note, among others, international health conferences during the 19^th^ or the formation of international health organisations in the 20^th^ century, as well as select initiatives such as the campaigns for eradicating malaria and smallpox, undertaken shortly before the constitution of the World Health Organization (WHO) [1-3].

It is in this spirit of bringing all people to the highest possible level of health that the WHO member countries, including several newly independent African ones, adopted the Alma Ata Declaration on primary health care in 1978 [4].

The Declaration makes health a human right and places it at the heart of social and economic development. It also emphasises the sovereign responsibility of governments for the health of their populations and underlines the importance of cooperation between all countries in a spirit of solidarity, with the goal to providing primary health care to their populations.

A little over four decades later, smallpox appears to have been eradicated. Nevertheless, malaria continues to decimate vulnerable populations in the Global South, particularly in sub-Saharan Africa [5], adding to the already growing burden of chronic noncommunicable diseases, accidents, respiratory diseases, and childhood pathologies, among others. This represents a considerable barrier to achieving sustainable human development.

In response, the WHO reiterated its call for a return to the Declaration of Alma Ata, as it represents a holistic and effective strategy for improving the health of populations and thus promoting sustainable human development [6-10].

Currently, we are facing worldwide, profound geostrategic and commercial transformations, as we have in past centuries. Countries of the Global South insist on sovereign development policies. Thus, the Brazil, Russia, China, India, and South Africa (BRICS) partnership should consider using the 1978 Alma Ata Declaration care within its framework for sustainable development.

Unlike current campaigns or initiatives based on selective short-term and hierarchically implemented interventions without the active participation of the target populations, primary health care, as specified by the seven principles of the Alma Ata Declaration, represents a holistic approach based on equity. The practice of primary health care requires the active participation of the communities concerned and intersectoral collaboration between all stakeholders. To a certain extent, one could clinically think of an effective development economics strategy [11]. For example, the Millennium Development Goals can be considered as a form of implementation of the Alma Ata Declaration; however, it is necessary to emphasise its selective aspect. Comprehensive, rather than selective primary health care might be able to respond adequately to the relationship between health and socioeconomic development [12].

The 14^th^ BRICS summit, held from 23 to 24 June 2022 in China, was marked by attention to the 2030 Sustainable Development Goals (SDGs). Specifically, the participants concernedly noted that the coronavirus disease 2019 (COVID-19) pandemic has disrupted efforts in achieving the 2030 Sustainable Development Agenda and reversed years of progress in social and economic development, but also environmental protection [13]

These concerns intensified during the 15^th^ summit held from 22 to 24 August 2023 in South Africa entitled “BRICS and Africa: Partnership for Mutually Accelerated Growth, Sustainable Development and Inclusive Multilateralism”. Its participants called for reform of the international economic institutions regarding specificities of emerging markets and developing economies, which should reflect their roles in the world economy. The BRICS countries also stressed the importance of beneficial economic growth and an effective fight against poverty in the countries of the Global South [14,15].

We cannot eradicate poverty, promote health, and promote human development by implementing the same selective, hierarchical strategies without including the target populations.

Having regained their independence in the early 1960s, the so-called Third World countries also reclaimed autonomy in stabilising their economies. However, they began to again lose it in favour of draconian structural adjustment measures led by the International Monetary Fund and the World Bank from the 1970s onwards [16]. These measures have been associated with intense pressures to further link these fragile economies to a dominant ultra-liberal economy.

The opponents of structural adjustment plans argue that high taxes, cuts in health and education, and the privatisation of key sectors of public life have contributed to increasing social inequalities and weakening the role of states in the process of human development [17-19]. It is in this spirit that UNESCO called for a global social adjustment in 1995 [16].

The BRICS Partnership for Sustainable Development and Inclusive Multilateralism recognises the sovereignty of governments as key in achieving sustainable human development. Its members reiterated the importance of multilateral cooperation and inclusively called for all national and international actors to implement corresponding reforms, recognising the importance of implementing the SDGs in an integrated and holistic way.

Nowadays, the 1978 Alma Ata Declaration seems to be the most appropriate approach. However, we must also take up the challenge of government sovereignty – more precisely, how governments can re-organise themselves sovereignly within the framework of the BRICS Partnership for Sustainable Development and Inclusive Multilateralism.

For this, the implementation of the federal law on primary prevention and on the promotion of health in the Federal Republic of Germany [20-23] can be used as an example. This law to strength health promotion and prevention came into force in Germany on 25 July 2015. It is intended to strengthen prevention and health promotion in settings and to promote cooperation between stakeholders at the federal state levels. The National Prevention Conference plays a key role in this context, which includes the delivery an agreement on nationwide framework recommendations for health promotion and prevention and a report with compulsory keys results on the development of health promotion and prevention every four years.

In the frame of their Partnership for Sustainable Development and Inclusive Multilateralism, countries of the Global South in general, and African countries in particular, could draw inspiration from this German model to sovereignly apply the 1978 Alma Ata Declaration.

In conclusion, just like the international health conferences or international organisations of past centuries, the BRICS can contribute effectively in the 21^st^ century to the promotion of health and human development by considering the 1978 Alma Ata Declaration within the framework of the Partnerships for Sustainable Development and Inclusive Multilateralism.
